# Growth Performance, Product Quality and Cost-Effectiveness of Alternative Protein Sources Replacing Fishmeal in *Penaeus vannamei* Reared in Biofloc Systems: Medium- and Large-Scale Studies

**DOI:** 10.3390/ani16182852

**Published:** 2026-09-10

**Authors:** S. Ferrando-Juan, J. Gómez-Aguilera, A. Tomás-Vidal, M. Rodilla, M. Jover-Cerdá, J. Calanche, S. Martínez-Llorens, D. S. Peñaranda

**Affiliations:** 1 Aquaculture and Biodiversity Research Group, Institute of Science and Animal Technology, (ICTA), Universitat Politècnica de València, Camí de Vera, s/n, 46022 València, Spain; sferjua@upv.es (S.F.-J.); jgomagu@posgrado.upv.es (J.G.-A.); atomasv@dca.upv.es (A.T.-V.); mjover@dca.upv.es (M.J.-C.); silmarll@dca.upv.es (S.M.-L.); 2 Research Institute for Integrated Management of Coastal Areas, Universitat Politècnica de València, Carretera de Nazaret-Oliva, s/n, Grao de Gandía, 46730 València, Spain; mrodilla@hma.upv.es; 3 Animal Production and Food Science Department, Faculty of Veterinary Medicine, AgriFood Institute of Aragón (IA2), Miguel Servet, 177, 50013 Zaragoza, Spain; calanche@unizar.es

**Keywords:** biofloc, shrimp, medium-scale, large-scale, alternative ingredients, fishmeal, replacement

## Abstract

Fishmeal (FM) reduction can improve shrimp feed sustainability, but low FM formulations remain insufficiently validated across production stages, scales, and post-harvest quality traits. This study evaluated alternative protein strategies for *Penaeus vannamei* reared under super-intensive biofloc technology (BFT) at different production scales. In a medium-scale grow-out trial, a Control diet containing 15% FM was compared with diets containing 7.5% FM using animal-derived proteins (A7.5) or combined animal- and plant-derived proteins (AP7.5). AP7.5 maintained growth and apparent feed conversion comparable to those of the Control diet, whereas A7.5 showed poorer performance. Post-harvest quality was assessed in commercial-size shrimp during refrigerated storage without antimelanosis treatment. Sensory differences were limited shortly after harvest but became more evident during storage, with AP7.5 maintaining a comparatively stable sensory profile and showing lower panel-assessed melanosis after seven days. Subsequently, the AP7.5 diet was validated against the Control in a pilot large-scale trial covering the juvenile and grow-out phases, maintaining comparable growth, economic conversion ratio, and profitability index. Sensitivity analysis further showed that its relative economic performance was maintained across simulated FM and shrimp price scenarios and became more favorable as FM prices increased. In conclusion, the consistent productive and economic performance of AP7.5 across production stages and scales, together with maintained sensory quality and lower panel-assessed melanosis, supports its potential as a scalable low-FM strategy for BFT shrimp production.

## Simple Summary

Reducing fishmeal in shrimp feeds can help make aquaculture more sustainable and cost-effective, but alternative diets must maintain shrimp growth, production efficiency and final product quality. This study evaluated reduced fishmeal diets for *Penaeus vannamei* reared under biofloc technology. In the AP7.5 diet, fishmeal inclusion was reduced from 15% to 7.5% by combining animal- and plant-based protein sources. Shrimp fed AP7.5 showed growth and feed utilization comparable to those fed the Control diet and greater economic efficiency than those fed the A7.5 diet. The productive performance of AP7.5 was also maintained in a larger pilot-scale trial covering different production stages, supporting its scalability under conditions closer to commercial production. After harvest, fishmeal reduction did not compromise sensory quality, and shrimp fed AP7.5 showed a comparatively stable sensory profile and lower melanosis after seven days of refrigerated storage. Overall, AP7.5 represents a practical low fishmeal strategy that maintained productive and economic performance across production scales while preserving post-harvest quality under biofloc conditions.

## 1. Introduction

The favorable nutritional properties of fishmeal (FM) have long established it as an ideal protein source in aquafeeds [1]. However, its use substantially affects feed costs and raises economic and environmental concerns related to the exploitation of small pelagic fish stocks and the ecological footprint of FM production [2,3]. Reducing FM inclusion therefore remains a major objective in shrimp nutrition. Considerable progress has been made in *Penaeus vannamei* (*P. vannamei*, Boone, 1931) using alternative protein sources, although responses to FM replacement may depend on shrimp size, stocking density, culture system, and experimental scale [4].

Alternative proteins differ considerably in their capacity to replace FM. Among plant-derived ingredients, rice protein concentrate maintained growth when FM was reduced from 46% to 23% [5], improved soybean meal (SBM) has been evaluated with FM progressively reduced from 30% to 0% [6], and poultry by-product meal allowed FM inclusion to decrease from approximately 39% to 8% while maintaining feed utilization and survival [7]. Therefore, successful FM reduction depends on both ingredient characteristics and the nutritional balance of the complete formulation. However, responses to FM replacement also depend on shrimp size, stocking density, and rearing conditions, and should be taken into consideration [4].

Regarding shrimp developmental stage and stocking density, it has been observed that during grow-out, a combination of SBM and peanut meal sustained growth and feed efficiency in *P. vannamei* of approximately 8.2 g when FM was reduced from 20 to 10%, whereas further reduction to 5% impaired both responses [8]. Similarly, a high SBM diet progressively reduced growth and poorer feed conversion was observed as density increased from 15 to 65 shrimp/m^2^ in outdoor tanks [9]. These findings indicate that FM-replacement strategies cannot necessarily be extrapolated across production stages and stocking conditions. Additionally, dietary protein requirements of approximately 38–47% crude protein (CP) have been reported for early juveniles, whereas around 30% CP can support satisfactory performance in larger shrimp [10,11,12].

The culture system may add another source of variability. Contrasting responses to alternative protein sources were reported in recirculating aquaculture system (RAS) and BFT trials [13]. For example, poultry by-product formulation performed least favorably in RAS, whereas the all-plant formulation showed the poorest response under BFT. These differences may arise because formulated feed represents the unique nutritional input in clear-water systems, while BFT provides an additional natural food source in the form of microbial aggregates within the bioflocs, which are directly consumed by shrimp. These aggregates supply protein, amino acids, lipids, minerals, and other bioactive compounds [14,15,16]. Stable isotope analysis has shown that biofloc can account for approximately 48–58% of assimilated nitrogen in juveniles and 37–42% during grow-out, with its contribution varying according to dietary protein supply [17]. It has been reported that biofloc can supplement diets restricted in essential amino acids and fatty acids [18], but its nutritional composition may be affected by diet formulation [19]. Beyond this direct nutritional contribution, digestive enzyme activity has generally been reported to be higher under BFT than under clear water conditions in shrimp fed FM-reduced diets [20]. Enhanced antioxidant responses have also been reported under BFT, including increased superoxide dismutase and glutathione peroxidase activity or expression [21,22]. Therefore, biofloc may modulate responses to alternative protein formulations, although an adequately balanced diet remains essential.

Experimental scale may further affect the transferability of dietary responses. Many studies supporting low FM formulations are based on small-scale trials with comparatively few validations performed under commercially relevant production conditions. Scaling up increases complexity in feeding management, biomass estimation, oxygen transfer, suspended solids, microbial activity, and nutrient dynamics. Accordingly, laboratory responses may not be reproduced with the same magnitude at commercial scale. For example, a marine resource-free formulation that performed comparably to conventional diets in laboratory trials subsequently resulted in improved feed conversion and greater final biomass than a commercial control in approximately 1000 m^3^ *P. vannamei* ponds [23], highlighting the importance of pilot-scale validation.

Our previous work showed that FM inclusion could be reduced by 50%, from 15% to 7.5%, using animal-derived proteins or a complementary animal and plant-derived protein sources without compromising juvenile *P. vannamei* performance under super-intensive BFT conditions (300 shrimp/m^3^) [24]. However, these findings have not yet been extended through grow-out to commercial size or validated for the most promising formulation in larger production units.

Dietary formulation may also influence post-harvest quality. Diet-induced changes in shrimp amino acid and fatty acid profiles do not always translate into perceptible sensory differences under BFT conditions [25,26,27]. Melanosis, a polyphenol oxidase (PPO) mediated process that reduces marketability, is commonly controlled using post-harvest antimelanosis treatments [28,29]. Dietary protein sources can modify free amino acids, trace minerals, polyunsaturated fatty acids, and antioxidant-related compounds in shrimp tissues [25,30]. These components may influence post-harvest deterioration through different pathways, but whether diet-induced changes alter intrinsic melanosis susceptibility, particularly when animal- and plant-derived proteins are combined, remains unknown.

Based on these knowledge gaps, the present study evaluated the productivity, nutritional composition and post-harvest product quality through medium- and pilot large-scale trials of selected low-FM diets in a previous work on juvenile *P. vannamei* reared under super-intensive BFT conditions [24]. In the current study, we hypothesized that: (i) a reduction in FM inclusion from 15% to 7.5% using an appropriate combination of animal- and plant-derived proteins would maintain growth parameters from juvenile to adult stage; (ii) the growth and economic performance of the selected low-FM formulation would remain consistent under pilot large-scale BFT conditions; and (iii) dietary protein source composition would influence sensory quality and melanosis development during refrigerated storage without antimelanosis treatment.

## 2. Materials and Methods

### 2.1. Ethics Approval

The use of *P. vannamei* in this study did not require approval from an institutional ethics committee, as crustaceans are not included within the scope of species covered under Directive 2010/63/EU [31]. However, all experimental procedures were designed in accordance with Royal Decree 53/2013 [32], Annex IV, and were considered to involve only mild interventions. Shrimp were euthanized using the cold thermal shock method in ice slurry, following the slaughter methodology established by [33]. The trial was conducted in collaboration between the Aquaculture Laboratory of the Universitat Politècnica de València (UPV; accreditation no. ES 46 250 0001091), where animal rearing and slaughter were performed, and the University of Zaragoza (UNIZAR), where the subsequent sensory analysis of the shrimp was conducted.

### 2.2. Experimental Design and Facilities

**Figure 1:**
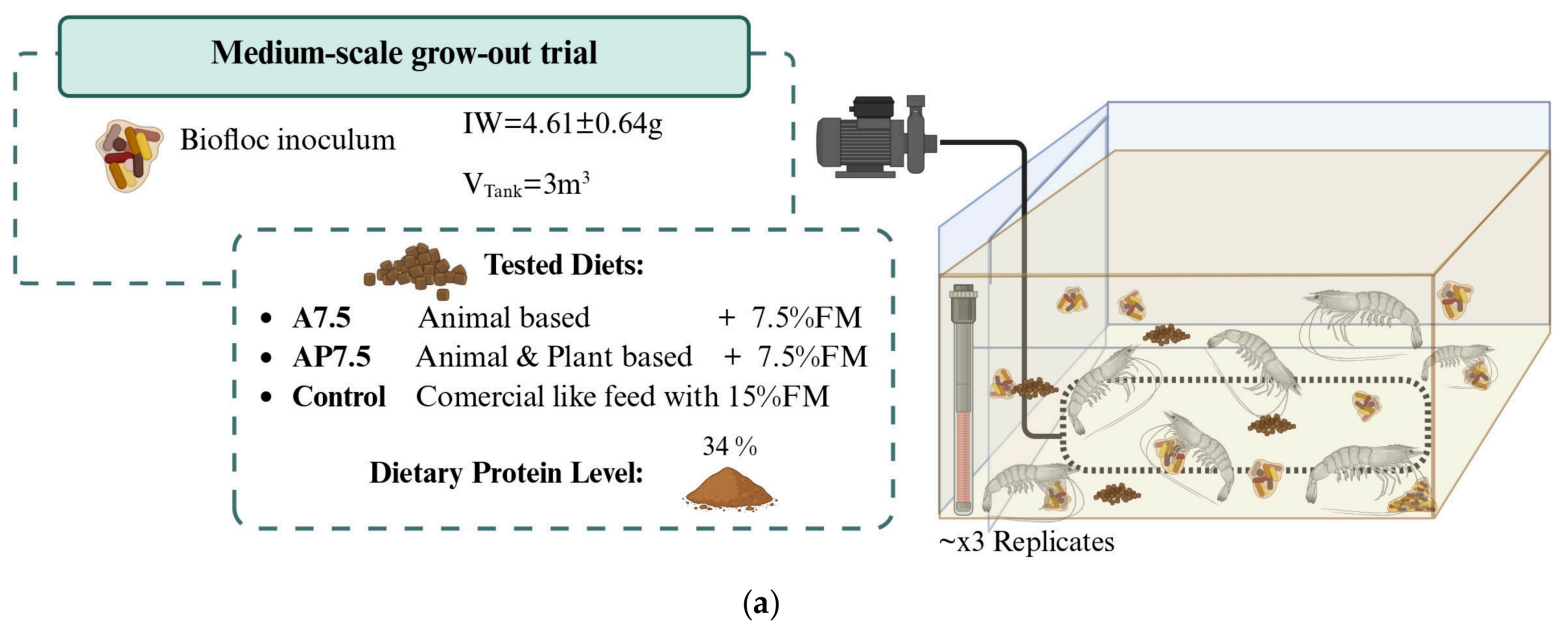

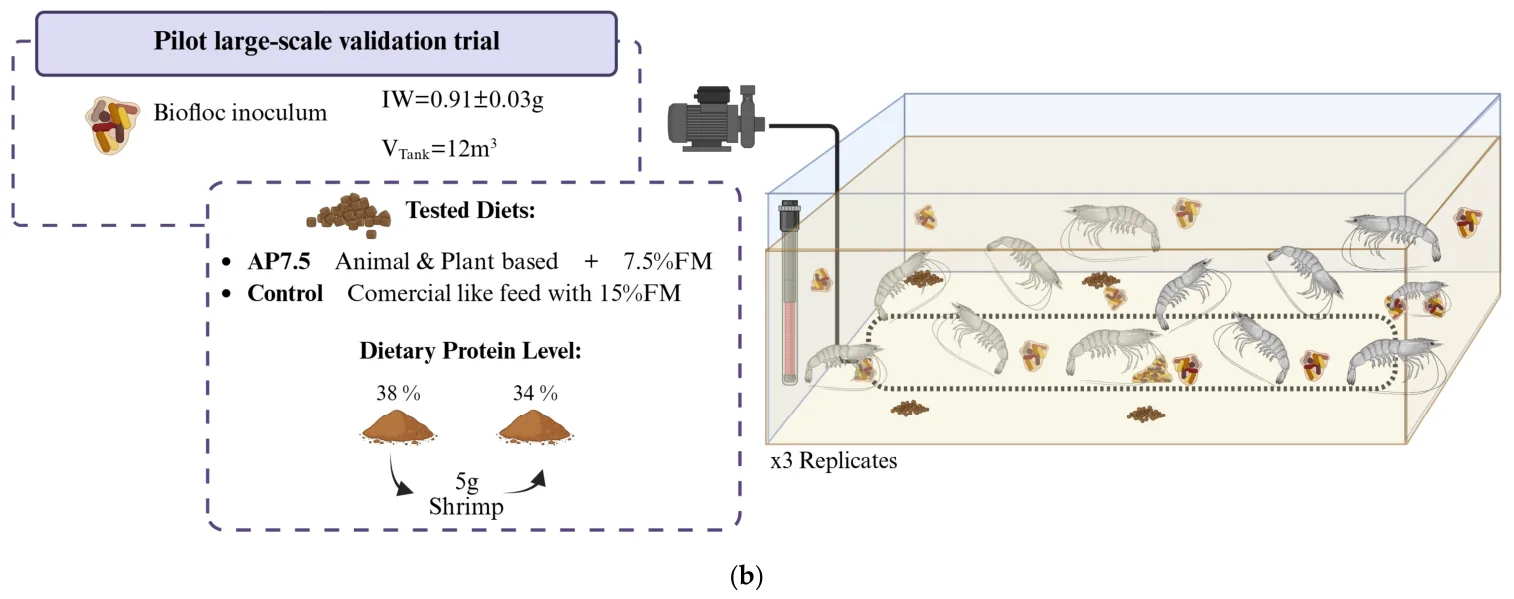
Experimental design diagram. (**a**) Medium-scale grow-out trial (3 m^3^), and (**b**) pilot large-scale validation trial (12 m^3^).

Two experimental diets, A7.5 and AP7.5 (Table 1), previously identified as promising strategies for FM reduction [24], were evaluated at medium and pilot scales to assess their performance in terms of shrimp growth, economic viability, and product quality. In a previous study, both diets allowed a FM reduction from 15 to 7.5% in the FM diet, without compromising the performance of juvenile *P. vannamei* (~2.5–8 g) [24]. In the present study, the A7.5 and AP7.5 diets were first evaluated against the Control diet (15% FM inclusion) during the grow-out phase (~4.5–15 g) in a medium-scale trial (Figure 1a). Based on the overall biological and economic performance observed in this trial, the best-performing experimental diet was subsequently compared with the Control diet in a pilot-scale validation trial covering the juvenile and grow-out stages under conditions closer to commercial production (Figure 1b).

For both trials, *P. vannamei* post-larvae (PL12) were supplied by White Panther (Edlach, Austria). After arrival, shrimp were gradually acclimated to 28 °C, alkalinity >150 mg/L, pH > 7.5, and dissolved oxygen >5 mg/L conditions and were fed commercial diets (LeGoussant Vanna PL and Vanna PreGrower) until reaching the target initial body weight for each trial. Shrimp were then randomly allocated to the experimental tanks. Throughout both trials, tanks were maintained under a 12:12 h light:dark photoperiod and equipped with continuous aeration through a Venturi system and individual heating units.

#### 2.2.1. Experimental Diets

The A7.5 and AP7.5 formulations were derived from a previous screening study in which alternative protein strategies based on animal (A), plant (P), or combined animal and plant (AP) sources were evaluated with 7.5% and 0% FM inclusion, together with a Control diet containing 15% FM [24]. A7.5 and AP7.5 were selected for further assessment because they maintained growth performance while showing favorable feed utilization responses. In the present study, both diets contained 7.5% FM, representing a 50% reduction relative to the Control diet. In A7.5, FM was partially replaced with animal by-products, specifically porcine hemoglobin and meat meal, whereas AP7.5 combined these animal-derived ingredients with plant protein sources, including hydrolyzed wheat meal, hydrolyzed soy protein, and potato protein.

Two dietary protein levels were formulated according to the production stage, based on previous results obtained for *P. vannamei* under BFT conditions [17]. Grow-out diets used in the medium-scale trial were formulated to contain 34% CP and 10% lipid. In the pilot large-scale trial, juvenile diets containing 38% CP and 10% lipid were provided until shrimp reached approximately 5 g body weight, followed by the corresponding grow-out diets containing 34% CP and 10% lipid.

All experimental diets were produced using an industrial twin-screw extruder (CLEXTRAL Evolum-25, Firminy, France) under standardized conditions. The extruder barrel consisted of five sections, with temperature settings of 70, 100, 100, 98–100, and 98–100 °C, respectively. Extrusion was performed at a pressure of 20–24 bar and a screw speed of 125–150 rpm. After extrusion, the feeds were dried at 30 °C in a forced-air circulation chamber (Airfrio, Almería, Spain) for 24 h and subsequently allowed to cool to room temperature. Finally, the feeds were stored at −20 °C until use.

Proximate composition was analytically verified prior to the trials. Ingredient composition and analyzed proximate composition are presented in Table 1, while amino acid profiles, determined after acid hydrolysis, are presented in Table 2 [34,35].

#### 2.2.2. Medium-Scale Grow-Out and Pilot Large-Scale Validation Trials

For the medium-scale trial, shrimp with an initial body weight of 4.61 ± 0.64 g were randomly distributed among nine 3 m^3^ tanks at a stocking density of 850 shrimp per tank (~300 shrimp/m^3^). Three replicate tanks were assigned to each dietary treatment (Control, A7.5, and AP7.5). Shrimp were reared under BFT conditions until they approached commercial harvest size (~15 g), with the trial lasting up to 71 days.

For the pilot large-scale validation trial, AP7.5 and the Control diet were evaluated in six 12 m^3^ tanks, with three replicate tanks per dietary treatment. Shrimp had an initial body weight of 0.91 ± 0.03 g and were stocked at 4200 shrimp per tank (~350 shrimp/m^3^). The trial lasted 62 days and comprised a 36-day juvenile phase, from approximately 0.9 to 5.5 g, followed by a 26-day grow-out phase. The corresponding juvenile and grow-out formulations described in Section 2.2.1 were provided during each phase.

### 2.3. Shrimp Growth and Feed Utilization

Shrimp were fed using automatic feeders, and feeding trays were used to verify actual feed intake and minimize overfeeding. Around 10% of the daily feed was supplied on the trays. When residual feed was detected, the daily ration for the subsequent day was reduced by 10% to maintain consistent feed management across all tanks. If residual feed was still detected at the subsequent inspection, the 10% reduction was maintained; otherwise, the planned ration was restored. The same feeding management protocol was applied to all experimental tanks, and rations were recalculated weekly based on biomass estimates obtained from growth sampling. As uneaten feed was not quantitatively collected, feed-use indices were calculated using the total feed offered rather than the amount effectively consumed. Accordingly, these indices should be regarded as apparent estimates, since a fraction of the supplied feed may have remained uneaten, been incorporated into the biofloc matrix, or been metabolized by the microbial community.

Growth performance was monitored weekly by randomly sampling shrimp from each experimental tank. In the medium-scale grow-out trial, approximately 12% of the animals were weighed, whereas around 5% were weighed in the pilot large-scale trial. These measurements were used to monitor growth over time, estimate tank biomass, and adjust feeding levels throughout the trials. In the medium-scale trial, feeding rates were adjusted according to a customized feeding table based on [36]. In the pilot large-scale trial, biomass estimation was less precise in the larger-volume tanks; therefore, ration adjustments were based on the practical recommendations described by [37]. The following zootechnical indicators were calculated:

[1] $Specific\;growth\;rate\left(SGR,\frac{\%}{day}\right)=100\times \frac{{ln}_{final\;weight}-{\;ln}_{initial\;weight}}{days}$;

[2] $Feed\;conversion\;ratio\;(apparent\;FCR)=\frac{feed\;supplied\;(g)}{biomass\;gain\left(g\right)}\;$;

[3] $Feed\;intake\;(FI,\frac{g}{100\;g\;shrimp\;day})=100\times \frac{feed\;supplied\;(g)}{average\;biomass\left(g\right)\times days}$;

[4] Protein intake ratio (PIR)=FI×dietary crude protein (%);

[5] $Protein\;efficiency\;ratio\;(PER)=\;\frac{Shrimp\;weight\;gain\;(g)}{total\;protein\;supplied\;(g)}$;

[6] $Survival\;(\%)=100\times \frac{Final\;nº\;of\;shrimp}{initial\;nº\;of\;shrimp}$.

### 2.4. Economic Analysis

The economic cost of each experimental diet was estimated based on the market price of the individual feed ingredients in December 2025, using an exchange rate of 1 USD = 0.868€. The complete list of ingredient prices used for the economic calculations is provided in the dataset deposited in the RiuNet institutional repository, as indicated in the Data Availability Statement.

An economic assessment of the diets was performed using the following indicators described by [38]:

[7] Economic conversion ratio (ECR; €/kg shrimp biomass produced) = FCR × diet price (€/kg);

[8] Economic profit index (EPI; €/kg biomass) = (final biomass × sale price) − feed cost.

The shrimp sale price used for calculations was 5.22 €/kg, based on [39].

To evaluate the robustness of the economic results to market variability, a scenario-based sensitivity analysis was performed by varying the baseline FM price (1.39 €/kg) and shrimp sale price (5.22 €/kg) by ±20% to represent plausible fluctuations around baseline market conditions, while keeping the experimentally observed biological performance and the prices of all other dietary ingredients constant [40,41]. ECR sensitivity to FM price was assessed in both the medium-scale and pilot large-scale trials, whereas the combined effects of FM and shrimp sale prices on EPI were evaluated for the pilot large-scale trial.

### 2.5. Shrimp and Biofloc Sampling

Biological samples were obtained at the initial and final stages of the medium-scale trial through random collection of shrimp and biofloc material. Specimens destined for proximate composition analysis were immediately preserved at −20 °C and, when required, subjected to freeze-drying prior to laboratory processing. For sensory quality and melanosis assessment, whole shrimp were stored under refrigeration at <2 °C and evaluated at 24 h and 7 days after slaughter. Biofloc samples intended for nutritional characterization were collected after allowing culture water to settle for 1 h; the resulting floc fraction was recovered and dried at 60 °C for 24 h before analysis.

### 2.6. Nutritional Composition of Shrimp and Biofloc

Proximate composition was determined according to standard analytical procedures. Dry matter (DM) content was quantified by oven-drying samples at 105 °C to constant weight, while ash content was obtained after incineration at 550 °C for 5 h, in accordance with 934.01 and 942.05 protocols [42], respectively. CP was estimated from total nitrogen using elemental analysis of nitrogen (N) and carbon (C) contents determined using a Leco CN628 analyzer (Leco Corporation, St. Joseph, MI, USA). Gross energy (GE) was subsequently calculated from carbon and nitrogen contents using the following equation:

[9] GE (MJ/kg) = 51.8 × C − 19.4 × N [43].

### 2.7. Water Quality Analysis

Physicochemical conditions in the biofloc system were controlled throughout the experiment. Temperature (°C), dissolved oxygen (mg/L), pH, and salinity (g/L) were recorded daily using a HANNA multiparameter probe (HI19829, Woonsocket, RI, USA) and a YSI ProODO meter (Yellow Springs, OH, USA). Total suspended solids (TSSs, mg/L) were quantified on a weekly basis using 0.45 µm Whatman glass-fiber filters [44], and excess sludge was removed whenever concentrations exceeded 500 mg/L. Alkalinity (mg/L) was determined twice weekly with a HANNA Checker photometer (HI755, Woonsocket, RI, USA). Concentrations of ammonium, nitrite, nitrate, and phosphate (NH_4_^+^, NO_2_^−^, NO_3_^−^ and PO_4_^3−^, mg/L) were analyzed by standard colorimetric procedures [45,46,47,48]. When necessary, pH and alkalinity were adjusted through the addition of calcium hydroxide and sodium bicarbonate.

Baseline water conditions were maintained at approximately 28 °C, salinity 21 g/L, alkalinity > 150 mg/L as CaCO_3_, pH > 7.5, and dissolved oxygen > 5 mg/L. In both experimental trials, biofloc development was promoted by inoculating the tanks with 125 mg/L of previously matured floc in disinfected seawater treated with 5% sodium hypochlorite. The system was operated without water exchange, and only freshwater was added periodically to compensate for evaporation losses, following the recommendations for zero-exchange BFT management [49].

### 2.8. Sensory Quality Profile and Melanosis Evaluations

Post-harvest quality assessments were conducted only on commercial-size shrimp harvested at the end of the medium-scale grow-out trial. A quantitative descriptive analysis (QDA) and an assessment of melanosis development were performed to evaluate the effect of dietary treatment on shrimp quality during refrigerated storage without antioxidant treatment. Both evaluations were conducted at one (24 h) and seven days post-slaughter. Each sample consisted of three whole shrimp, presented either raw or steamed, unseasoned, and at room temperature. The visual attributes of color, brightness, and melanosis were evaluated in both raw and cooked shrimp, while the remaining sensory attributes were assessed only in cooked shrimp.

All quality evaluations were conducted by trained expert panelists comprising 14 assessors in the sensory analysis room of the Pilot Plant at UNIZAR, following ISO 13299:2016 standards [50]. Assessors received prior training in the use of the score sheets and intensity scales in accordance with UNE-EN ISO 8586:2014 [51]. All assessors had previously demonstrated high accuracy and reproducibility in sensory analyses and possessed well-developed long-term sensory memory, ensuring consistent and reliable comparative judgments.

For the QDA, structured scales anchored at the extremes for specific sensory attributes identified for shrimp were employed [52]. The evaluation score sheet is provided in the Supplementary Material, Annex SI. Based on the results obtained, β coefficients were calculated for both time points, and a within-group (intra-lot) analysis was performed. In this analysis, values above the x-axis indicate the presence or intensification of the evaluated sensory attributes, whereas values below the axis reflect their absence or reduction. Sensory descriptors marked with an asterisk (*) indicate statistically significant differences for that specific attribute as perceived by the panelists. Adjusted mean values per product were then used to construct sensory profiles. An inter-group (inter-lot) analysis was also performed to compare the different treatments and evaluate sensory changes over time. Finally, a principal component analysis (PCA) was conducted based on the sensory profiles, and the results were visualized using a biplot representation.

Melanosis was evaluated following the methodology described for shrimp [53], and using a predefined specialized evaluation scale adapted for raw and cooked *P. vannamei* [54]. The same standardized scoring criteria were applied by all assessors. The evaluation score sheet is provided in the Supplementary Material, Annex SII.

To assess the reliability of the melanosis scores, panel performance was evaluated using the Sensory Panel Performance tool implemented in XLSTAT (Lumivero, Denver, CO, USA, 2026). Panel discrimination, repeatability, agreement, and stability were assessed using ANOVA and the Control of Assessor Performances (CAP) procedure. Scale use and inter-assessor consensus were further examined using graphical and multivariate approaches, including PCA and MFA, while Euclidean distances, Wilcoxon tests, clustering, and STATIS analyses were used to identify atypical assessors, session effects, and overall panel homogeneity. This procedure provided an objective statistical assessment of panel performance and the reliability of the sensory evaluations.

### 2.9. Statistical Analysis

Data obtained from shrimp performance indicators, proximate composition analyses, water quality variables, and sensory evaluations were analyzed using Statgraphics Centurion 18 software (StatPoint Technologies, Warrenton, VA, USA). Tanks were considered the experimental units. In the medium-scale grow-out trial, growth and feed utilization variables were analyzed by analysis of covariance (ANCOVA), using initial mean body weight as a covariate to account for variability among experimental units. For the remaining variables and those from the pilot large-scale trial, data suitability for parametric analysis was assessed using the Shapiro–Wilk test, skewness and kurtosis, and normal probability plots. When parametric assumptions were met, treatment effects were evaluated by one-way ANOVA followed, when significant, by the Newman-Keuls multiple-range test. Otherwise, the Kruskal–Wallis test was applied, followed by Bonferroni-adjusted pairwise comparisons.

To assess whether the growth response to dietary treatment varied throughout the pilot large-scale trial, body weight over time and phase specific SGR were further analyzed by repeated measures ANOVA, with Diet (AP7.5 and Control) as the between subject factor and tank as the experimental unit (*n* = 3). For body weight, Time was included as the repeated factor. For SGR, production phase (juvenile and grow-out) was used as the repeated factor. Greenhouse-Geisser correction was applied to the within-subject effects of the shrimp body weight analysis. Statistical significance was accepted at *p* < 0.05.

## 3. Results

### 3.1. Shrimp Performance

#### 3.1.1. Growth Parameters

In the medium-scale grow-out trial, the diets combining animal and plant-based protein sources resulted in better growth parameters, particularly in terms of SGR and apparent FCR. These results show that the AP7.5 formulation was able to maintain growth performance comparable to that of the Control diet, whereas the A7.5 diet was not able to compensate for the FM substitution. In the pilot large-scale trial, AP7.5 maintained similar growth performance to the Control diet (Table 3). Thus, the comparable performance obtained with AP7.5 during the medium-scale trial was confirmed under larger-scale production conditions.

**Figure 2:**
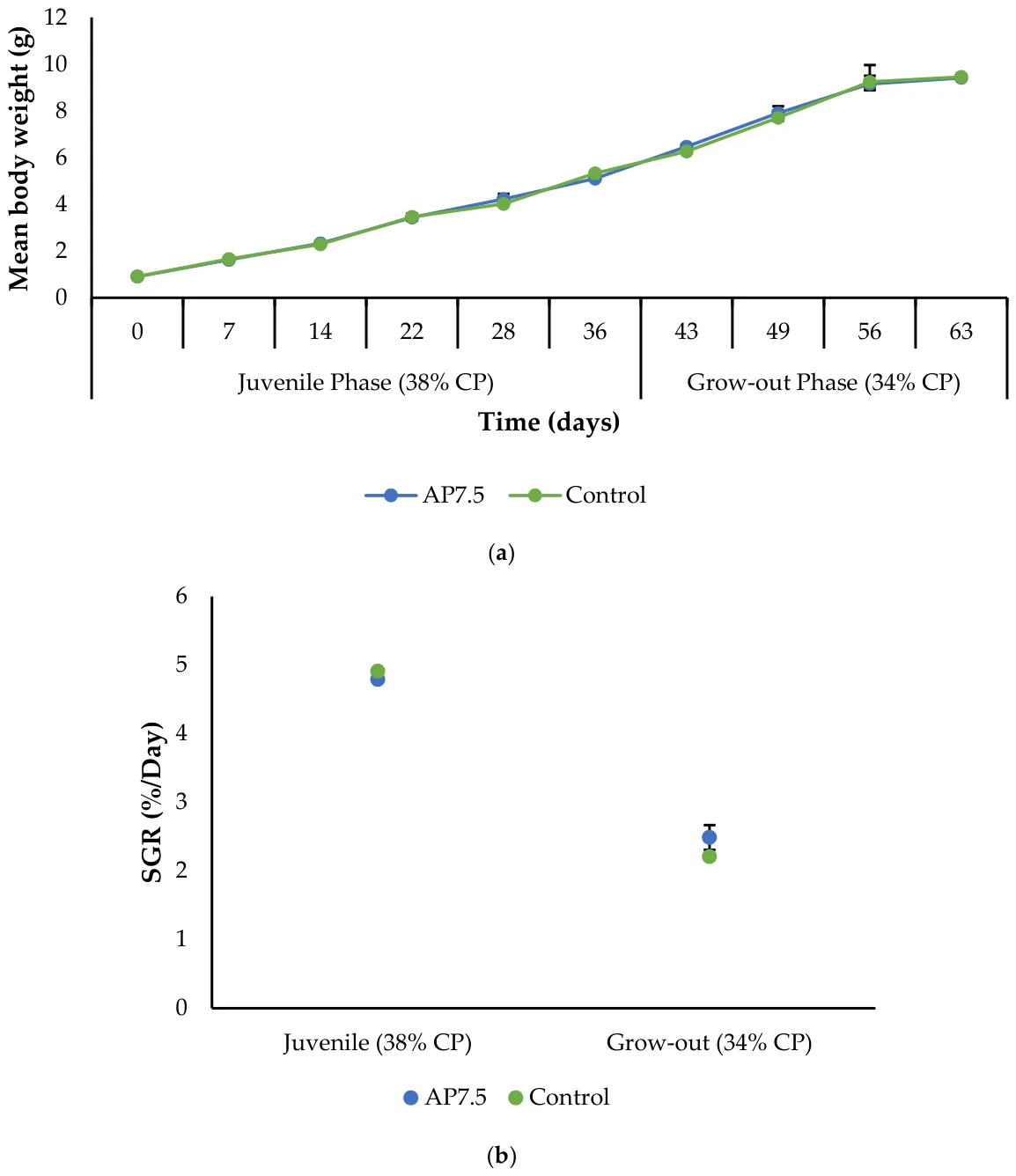
Growth response of *P. vannamei* fed AP7.5 and Control diets during the pilot large-scale validation trial. (**a**) Mean body weight evolution throughout the juvenile (38% CP) and grow-out (34% CP) phases. (**b**) Phase-specific growth rate (SGR) (mean ± SE; *n* = 3).

Repeated measures ANOVA of shrimp body weight in the pilot large-scale trial further showed that body weight increased significantly over time, whereas neither Diet nor the Diet × Time interaction was significant (Figure 2a; Supplementary Material, Annex SIII, Table S1). Thus, AP7.5 and the Control followed comparable growth trajectories throughout the juvenile and grow-out phases. Consistently, SGR decreased significantly from the juvenile to the grow-out phase, while neither Diet nor the Diet × Phase interaction was significant (Figure 2b; Supplementary Material, Annex SIII, Table S1). Together, these results indicate that the relative growth response to AP7.5 remained consistent across the two production phases evaluated in the pilot large-scale trial.

#### 3.1.2. Economic Analysis

In the medium-scale grow-out trial, AP7.5 achieved an ECR comparable to the Control diet and significantly lower than A7.5 (Table 4). The lower ECR of AP7.5 relative to A7.5 reflected its more efficient feed conversion rather than a lower diet price, indicating that its improved feed use efficiency translated into lower feed-related biomass production costs. This difference was consistent with the higher feed conversion efficiency observed for AP7.5, which translated into improved production economics relative to A7.5. Accordingly, the use of the AP7.5 and Control diets reduced production costs by approximately 0.89 € per kg of biomass compared with A7.5.

During the pilot large-scale validation trial, ECR values remained similar between treatments, regardless of the protein level evaluated (38% CP and 34% CP), resulting in comparable overall ECR values. Likewise, EPI did not differ significantly between treatments. These results show that the growth performance maintained with AP7.5 at the large-scale trial was accompanied by economic performance comparable to that of the Control diet despite the 50% reduction in FM inclusion.

Sensitivity analysis further showed that the economic performance of AP7.5 was robust to the simulated variations in FM and shrimp market sale prices (Supplementary Material, Annex SIII, Tables S2–S4). Across FM price scenarios, AP7.5 maintained a lower ECR than A7.5 and remained comparable to the Control in the medium-scale trial, while maintaining a lower overall ECR than the Control in the pilot large-scale trial. In both trials, the relative economic performance of AP7.5 became more favorable as FM prices increased. Likewise, AP7.5 retained a numerically higher EPI than the Control diet across all nine FM and shrimp price combinations evaluated. Changes in shrimp sale price affected absolute profitability but did not alter the relative economic ranking of the two diets.

#### 3.1.3. Effect of Diet on Water Quality

Water quality remained within the recommended limits for *P. vannamei* culture throughout the experimental period. During the medium-scale grow-out trial, dissolved oxygen, salinity, and alkalinity showed statistically significant differences among treatments; treatment-specific values and statistical comparisons are provided in Supplementary Material, Annex SIII, Table S5. However, these differences were sporadic, showing no consistent treatment-related pattern. Moreover, they were not accompanied by treatment-related differences in final weight or survival, indicating that culture conditions remained suitable across dietary treatments.

#### 3.1.4. Nutrient Utilization and Proximate Composition of Shrimp and Biofloc

Protein and energy retention efficiencies did not differ significantly among dietary treatments during the medium-scale grow-out trial (Table 5), indicating that the tested dietary formulations did not significantly affect whole-body composition.

Similarly, dietary treatments did not affect shrimp or biofloc composition (Table 6). However, significant differences between the initial and final sampling times were observed in both shrimp and biofloc. Shrimp showed increased DM, CP, and energy contents over time, whereas biofloc exhibited lower OM and higher ash contents at the end of the trial than at the initial sampling.

### 3.2. Post-Harvest Quality and Shelf-Life Assessment

#### 3.2.1. Sensory Evolution

Sensory changes were evaluated using two complementary approaches: an intra-lot analysis to characterize the temporal evolution of each dietary group and an inter-lot analysis to compare treatments at each storage time.

**Figure 3:**
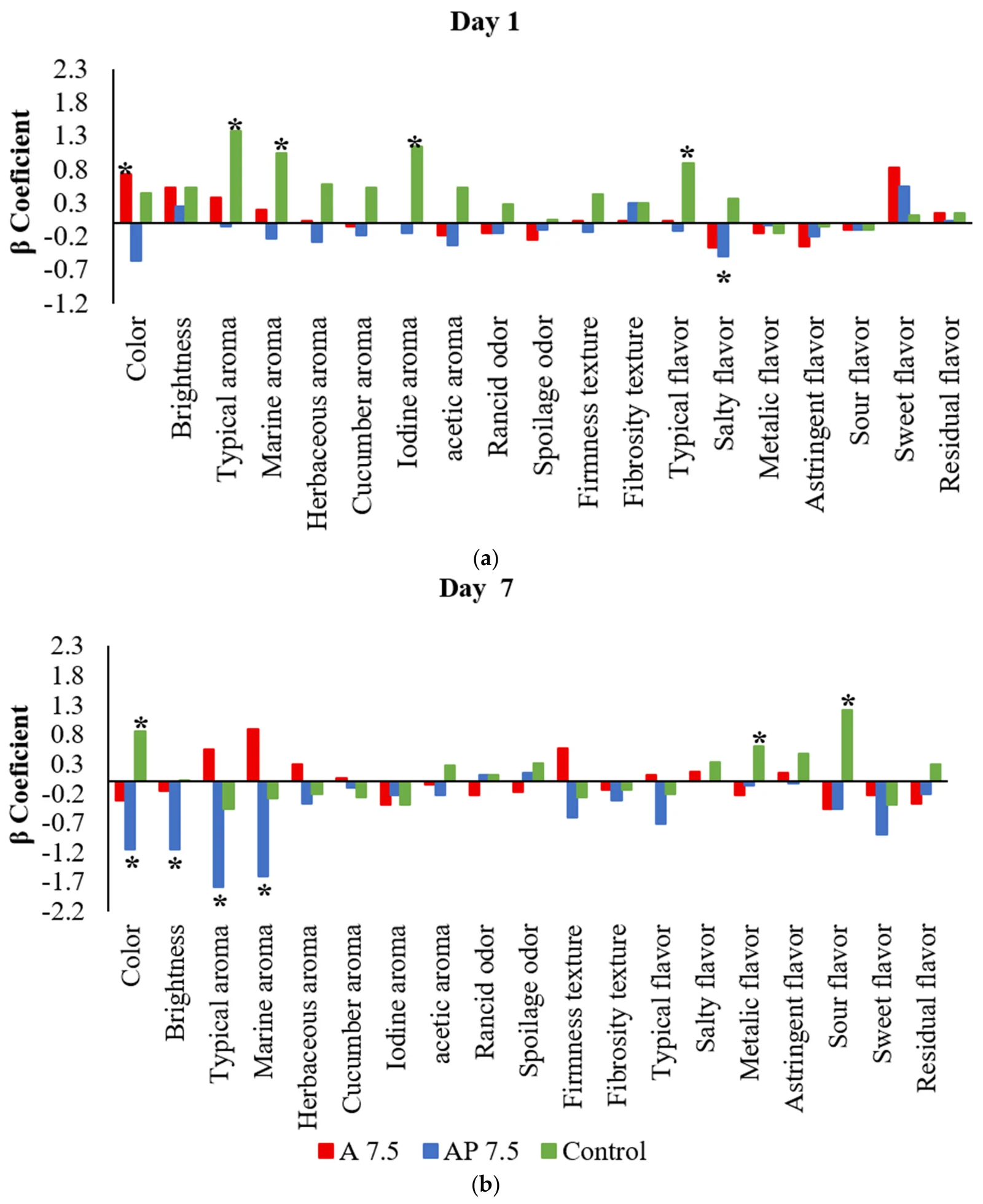
β coefficients obtained for the sensory descriptors evaluated in shrimp fed on three diets (A7.5, AP7.5 and Control) at one (**a**) and seven (**b**) days post-slaughter, based on the intralot analysis. (*) symbol represents a significant difference between sensory descriptors in each treatment (*p* < 0.05).

The β coefficients obtained from the intra-lot analysis at one and seven days post-slaughter are shown in Figure 3a,b. At day one (24 h post mortem), shrimp fed the Control diet displayed the most pronounced characteristic sensory profile, particularly for typical, marine, and iodine aromas, as well as a strong typical flavor. However, by day seven, this profile shifted toward metallic and sour flavor notes, while color remained relatively pronounced. Shrimp fed the A7.5 diet also showed a distinct initial profile, especially for color, but the intensity of its sensory descriptors decreased during storage, resulting in the absence of distinctive characteristics at later storage stages. In contrast, shrimp fed the AP7.5 diet exhibited a less pronounced sensory profile at the beginning of storage, characterized by lower intensity of typical marine-related descriptors. This relatively neutral profile remained stable over time, without the clear development of dominant positive or negative sensory attributes during refrigerated storage. These results indicate that sensory profiles evolved differently among dietary treatments.

**Figure 4:**
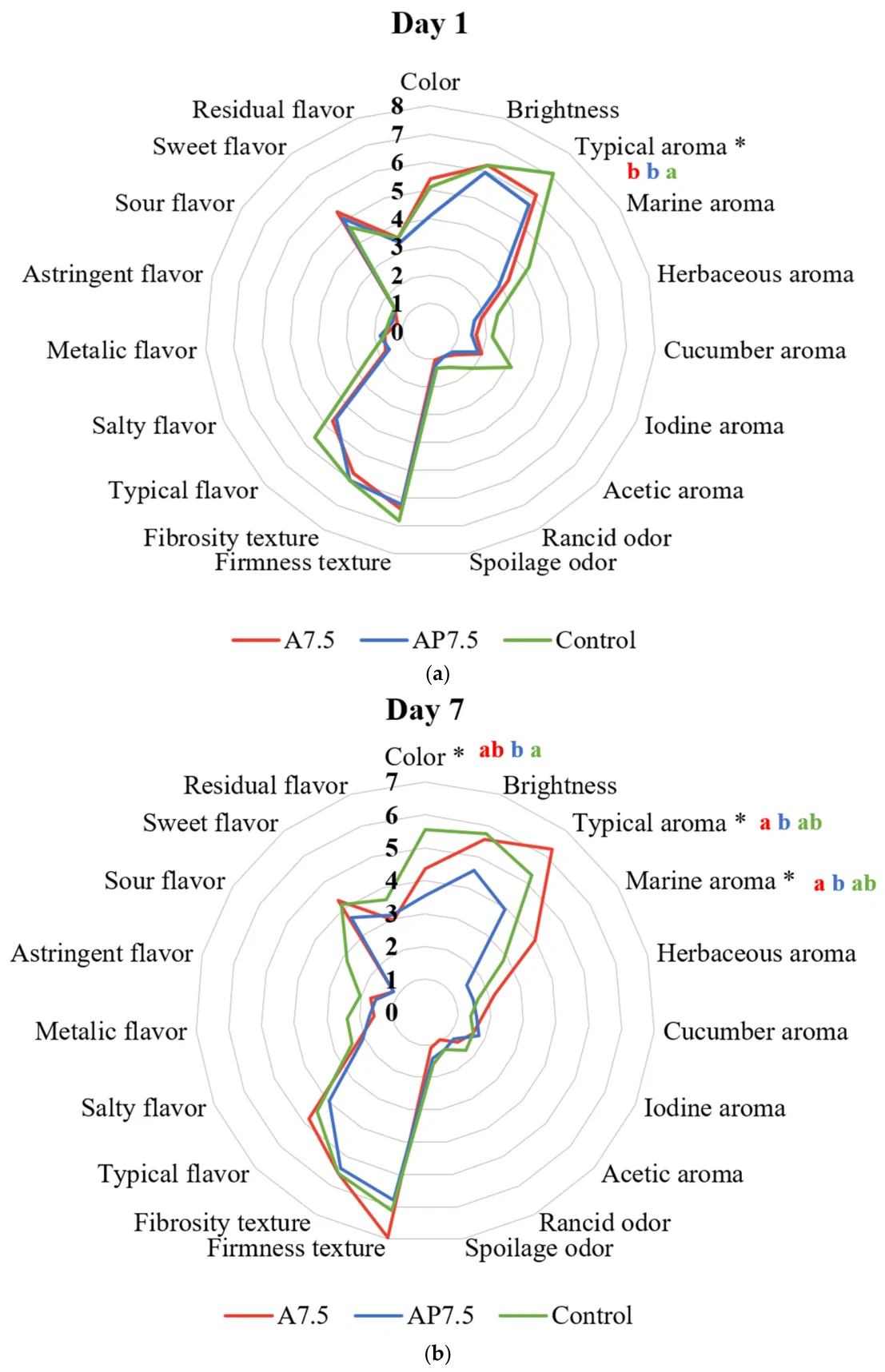
Sensory profile of shrimp fed three diets (A7.5, AP7.5, and Control), evaluated at one (**a**) and seven (**b**) days post-slaughter, based on the inter-lot analysis. (*) symbol represents a significant difference between treatments (*p* < 0.05). Lowercase letters denote statistically significant differences between treatments with a significance level of *p* < 0.05.

The inter-lot analysis further identified treatment-specific differences at each storage time (Figure 4a,b). On day one, the only discriminating attribute was the typical shrimp aroma, with higher scores observed in the Control group compared with alternative diets, while the remaining attributes showed similar values across treatments. By day seven, treatment differentiation became more evident, particularly for color and marine-related aroma descriptors. The Control group showed the highest color scores, although values were not significantly different from those of A7.5, whereas A7.5 tended to show higher marine and typical aroma scores.

**Figure 5:**
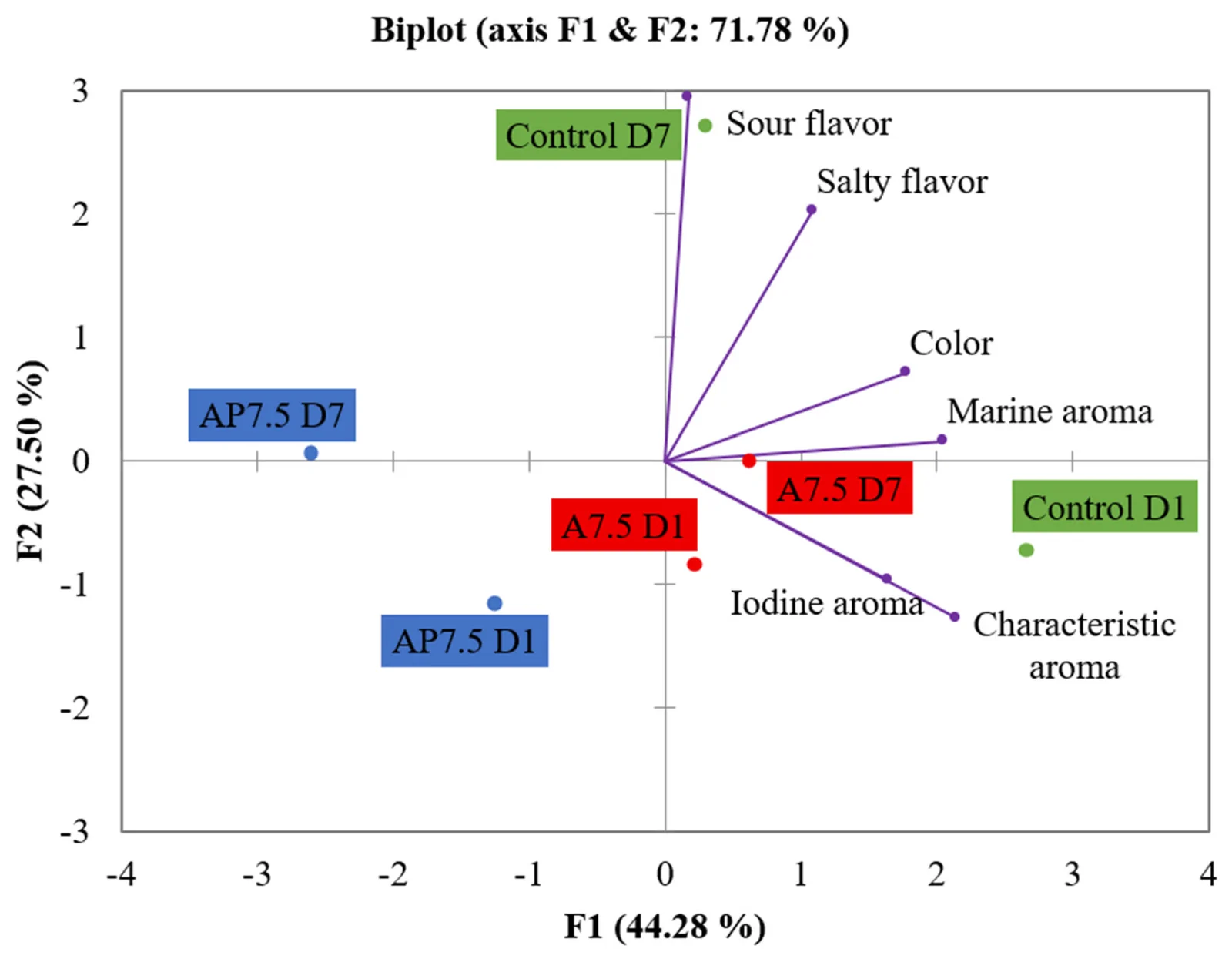
PCA plot of the sensory profile of shrimp fed three diets (A7.5, AP7.5, and Control), evaluated at one and seven days post-slaughter, along with the statistically significant sensory descriptors.

To integrate these results, a PCA was performed (Figure 5), which explained 72% of the total variance. Shrimp fed the AP7.5 diet were weakly associated with the evaluated sensory vectors, consistent with their comparatively neutral sensory profile. In contrast, shrimp fed the Control and A7.5 diets showed clearer associations with specific descriptors. At day one post-slaughter, shrimp from both the control and A7.5 treatments were associated with typical and iodine aromas, whereas on day seven, the Control diet was related to salty and sour flavors, and A7.5 to marine aroma and color. Overall, the multivariate analysis further supported the finding that dietary effects on the sensory profile became more evident during refrigerated storage.

#### 3.2.2. Melanosis Evaluation

**Figure 6:**
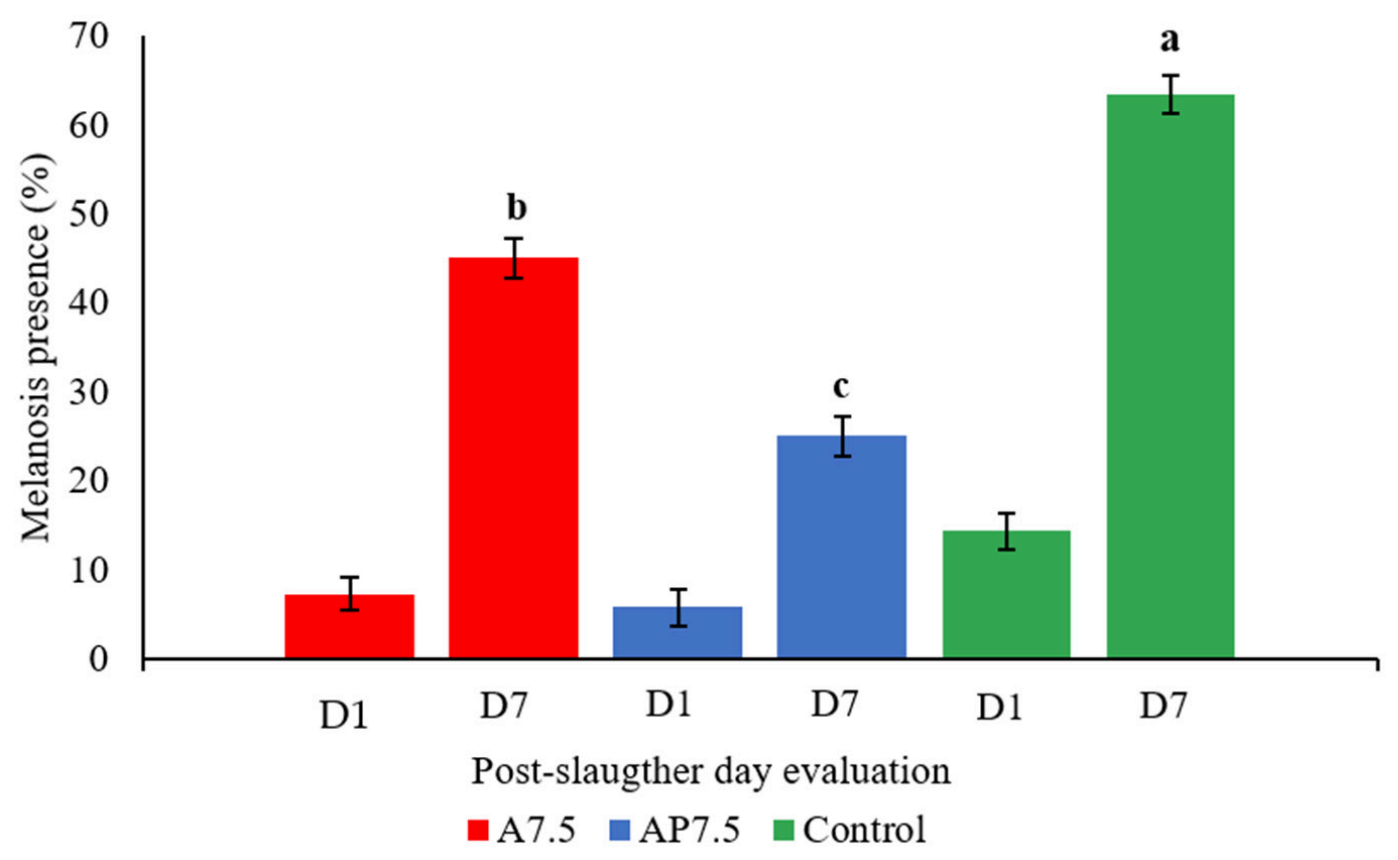
Presence of melanosis in the shrimp fed with three different diets (A7.5, AP7.5, and Control), evaluated at one and seven days post-slaughter. Lowercase letters represent significant differences between groups (*p* < 0.05).

At the end of the medium-scale grow-out trial, melanosis incidence averaged approximately 9% and did not differ significantly among dietary treatments after one day post-slaughter. After seven days, clear differences among dietary groups were detected. Shrimp fed the AP7.5 diet showed the lowest melanosis incidence (25%) and were not rejected by the sensory panel. In contrast, shrimp from the A7.5 and Control treatments exhibited significantly higher levels than AP7.5 shrimp (45% and 63%, respectively) and were rejected by the panel (Figure 6). These results indicate that dietary formulation influenced the progression of melanosis during storage, with AP7.5 showing a lower susceptibility than the other treatments, suggesting a potential influence of dietary composition on post-harvest quality traits under BFT conditions.

### 3.3. Cross-Scale Consistency of AP7.5 Performance

An integrated comparison of AP7.5 performance across experimental scales is presented in Table 7. Despite differences in shrimp size and production scale, the relative response of AP7.5 compared with the Control diet remained consistent for growth, feed utilization, survival, and economic conversion in both trials. AP7.5 maintained SGR and apparent FCR close to those of the Control diet in both trials, while its relative ECR was comparable in the medium-scale trial and numerically lower in the pilot large-scale trial. Relative differences were generally small and followed a consistent pattern across scales, supporting the reproducibility of the productive and economic performance of AP7.5 under the evaluated conditions. Post-harvest melanosis was assessed only in the medium-scale trial and EPI in the pilot large-scale trial; therefore, variables were not included in the cross-scale validation.

## 4. Discussion

The present study showed that FM inclusion can be reduced from 15% to 7.5% in *P. vannamei* reared under BFT across production stages and experimental scales. AP7.5 diet maintained productive and economic performance comparable to the Control diet across the evaluated conditions and was associated with lower post-harvest melanosis in commercial-size shrimp. In the medium-scale grow-out trial, shrimp fed AP7.5 maintained SGR and apparent FCR values comparable to those obtained with the Control diet, whereas A7.5 resulted in lower SGR and poorer feed conversion. Because both experimental diets contained the same FM inclusion level, these contrasting responses indicate that the effectiveness of FM reduction depended primarily on the composition and nutritional complementarity of the alternative protein sources used in the formulation.

This formulation-dependent response may be partially explained by differences in the nutritional complementarity of the protein sources used in each formulation [1]. Although the three grow-out diets showed relatively similar overall proportions of essential amino acids (EAA; 41.4–42.9% of total amino acids) and EAA/NEAA ratios (0.71–0.75), differences were evident in the distribution of individual EAA. In particular, the amino acid profile of AP7.5 more closely resembled the Control diet than did A7.5 for several essential amino acids, including isoleucine, lysine, methionine, phenylalanine, and threonine. This is consistent with the broader diversity of protein sources in AP7.5, whereas A7.5 relied more heavily on porcine hemoglobin and meat meal. Combining protein sources with different amino acid profiles may therefore have reduced nutritional imbalances associated with individual ingredients and contributed to the more efficient feed utilization observed with AP7.5.

The importance of ingredient selection is also supported by previous FM replacement studies in *P. vannamei*, showing that productive responses depend on the characteristics, inclusion levels, and processing of the alternative ingredients used. Moderate FM replacement with animal-derived proteins can be successfully achieved, whereas excessive inclusion levels (>80%) may impair growth performance and feed efficiency depending on the ingredient considered [55,56]. Moreover, derived blood ingredients may require particular consideration, as hemoglobin powder has also been associated with reduced growth performance at comparatively moderate inclusion levels [57]. These observations are consistent with the poorer performance of the animal protein-dominated A7.5 formulation and further support the potential advantage of combining ingredients with complementary nutritional characteristics. Nevertheless, because amino acid digestibility and availability were not directly determined in the present study, the contribution of specific amino acids to the contrasting responses of A7.5 and AP7.5 cannot be established.

These ingredient-level effects, however, should also be interpreted accounting for the nutrient contributions of the biofloc community. Despite marked differences in the amino acid and fatty acid profiles of feeds with varying FM inclusion, those of biofloc and shrimp have been reported to remain comparatively stable under partial FM replacement, suggesting a potential compensatory contribution of biofloc-derived nutrients [20]. Similarly, microbial floc can contribute nutritionally to *P. vannamei* even when shrimp are fed diets constrained in essential amino acids and fatty acids [18]. However, this additional trophic pathway does not appear to eliminate the importance of dietary formulation. In the present study, protein and energy retention efficiencies and shrimp proximate composition were not significantly affected by diet despite the poorer SGR and apparent FCR observed with A7.5. This combination of responses is compatible with a buffering contribution of biofloc derived nutrients, while the lower feed use efficiency of A7.5 indicates that such supplementation was insufficient to fully overcome differences among dietary protein source combinations. The culture environment may therefore modulate, but not override, the nutritional consequences of FM replacement, consistent with the different relative responses to alternative protein formulations reported in independent clear water and BFT trials [13]. This nutritional buffering provides a plausible explanation for why the poorer response to A7.5 was mainly reflected in SGR and apparent FCR, without changes in nutrient retention efficiency or shrimp body composition.

Against this nutritional background, the pilot large-scale validation trial provides evidence that the AP7.5 diet maintained performance comparable to the Control as shrimp progressed from the juvenile to the grow-out size and culture conditions became more production-relevant. Stage-specific adjustment of dietary protein is consistent with previous BFT studies. Diets containing 40% CP during nursery and 38% CP during grow-out have been successfully used in *P. vannamei*, while the nutritional contribution of biofloc was reported to vary with shrimp size [58]. More specifically, our previous study identified 38% CP as an efficient dietary level for juveniles, whereas 34% CP was sufficient to sustain growth during grow-out under BFT conditions. Stable-isotope analysis further showed that biofloc accounted for approximately 48–58% of assimilated nitrogen in the juvenile treatments included in the isotopic analysis and 37–42% during grow-out [17]. In the present study, dietary CP was reduced from 38% during the juvenile phase to 34% during grow-out. Despite this stage-specific adjustment, AP7.5 maintained performance comparable to the Control throughout both production phases. The absence of significant Diet × Time and Diet × Phase interactions further supports the consistency of this response, suggesting that the animal and plant protein strategy remained effective under both nutritional regimes. Moreover, the productive performance achieved in the 12 m^3^ tanks was consistent with that reported in other pilot-scale BFT studies; shrimp stocked at approximately 0.9 g in 10 m^3^ tanks, for example, reached 10.1–11.5 g after 78 days, with survival of 75.9–88.2% and FCR values of 1.6–1.7 [59]. Together with the comparable performance previously observed in pre-juvenile shrimp, these findings extend previous evidence supporting AP7.5 by validating the formulation across successive production stages and under larger, more production-relevant conditions [24].

The economic response further supports the practical applicability of AP7.5. Its lower ECR than A7.5 at medium scale was primarily driven by improved feed conversion rather than by differences in diet cost, while ECR and EPI remained comparable to the Control in the pilot large-scale trial. Interestingly, ECR was numerically lower at the pilot scale than at the medium scale for both AP7.5 and the Control diet, suggesting more efficient feed-related biomass production under the larger-scale conditions evaluated. This trend is consistent with reports of increasing returns to scale in *P. vannamei* farming and with the potential economic benefits of combining larger production volumes with intensive BFT operation [60,61]. Importantly, the relative economic position of AP7.5 was maintained when FM and shrimp prices were varied in the sensitivity analysis and became progressively more favorable as FM price increased. Therefore, reducing FM from 15% to 7.5% did not compromise economic performance and may provide greater economic resilience as FM prices increase.

Beyond growth and economic performance, the present study evaluated the impact of dietary formulation on post-harvest quality of commercial-size shrimp stored under refrigeration without antimelanosis treatment. Across all treatments, desirable sensory attributes were maintained, and deterioration-related descriptors remained low, supporting the ability of BFT to produce high-quality shrimp [33]. Nevertheless, dietary treatments exhibited different sensory trajectories during storage. Differences were limited shortly after harvest but became more evident during refrigerated storage, suggesting that protein source composition influenced the evolution of sensory attributes rather than markedly altering initial shrimp quality. This pattern was evident both in the temporal evolution within each dietary group and in the progressive differentiation among treatments. The temporal analysis showed distinct sensory trajectories among treatments, with the Control and A7.5 exhibiting greater changes in descriptor intensity over storage, whereas AP7.5 maintained a comparatively more stable and neutral sensory profile. Consistently, comparisons among dietary groups showed only limited differentiation at day one, mainly associated with typical shrimp aroma, whereas differences in color and marine-related aroma became more evident after seven days. The PCA further supported this interpretation showing a balanced sensory profile for AP7.5 for the evaluated sensory descriptors. These complementary responses indicate that substantial FM reduction did not compromise the overall sensory quality of shrimp, but that the combination of protein sources modulated how sensory characteristics evolved after harvest. This relatively limited effect of diet immediately after harvest agrees with previous studies in *P. vannamei*, in which substantial FM replacement or changes in dietary protein were not associated with major impairment of sensory quality or acceptance [26,27]. However, the increasing differentiation among treatments during storage suggests that dietary effects may become more evident as post-mortem changes progress. This interpretation is supported by evidence that dietary formulation can modify muscle compounds directly involved in taste and aroma, including inosine monophosphate, flavor-related amino acids, purine metabolites, fatty acids, and volatile compounds [25,62]. Such compositional changes may initially have a limited sensory impact but become more relevant during refrigerated storage as nucleotide degradation, lipid oxidation, and volatile-compound formation progress. In addition, changes in flesh quality have been observed after FM replacement even when growth performance was maintained, indicating that productive responses and post-harvest quality do not necessarily vary in parallel [30]. Therefore, the different sensory trajectories observed among treatments may reflect diet-induced differences in muscle composition that became progressively expressed during post-mortem storage. Nevertheless, because these compounds and their temporal evolution were not quantified in the present study, their specific contribution to the observed sensory differences cannot be established.

Dietary formulation was also associated with differences in melanosis development during refrigerated storage, an important aspect of shrimp visual quality and marketability. Current strategies to control melanosis are predominantly applied after harvest and include sulphites, 4-hexylresorcinol and other synthetic or natural antimelanotic treatments [63,64,65]. In contrast, the potential influence of pre-harvest nutritional strategies on subsequent melanosis development remains comparatively underexplored. Although some attempts have been made to incorporate antimelanotic compounds directly into shrimp diets, supplementation with an ergothioneine-rich mushroom extract, for example, has been shown to reduce PPO activity and delay post-mortem melanosis development [66]. However, evidence regarding the influence of feed composition itself on post-harvest melanosis remains limited. The present study did not include specific antimelanotic compounds, but evaluated whether the composition of the protein sources used in the diets was associated with subsequent product stability. Under these conditions, AP7.5-fed shrimp showed substantially lower panel-assessed melanosis after seven days of refrigerated storage. Although melanosis was evaluated by trained panelists using a standardized shrimp-specific scale, complementary biochemical and instrumental measurements, including PPO activity, melanin content, antioxidant-related compounds, or shell L*ab* values, were not obtained. Consequently, the mechanisms underlying the lower melanosis observed in AP7.5-fed shrimp cannot be established from the present study, and the response should be interpreted specifically as a difference in panel-assessed melanosis rather than as evidence of a particular antioxidant or biochemical mechanism. Because post-harvest quality was evaluated only in commercial-size shrimp harvested at the end of the medium-scale trial, the lower panel-assessed melanosis observed with AP7.5 cannot be extrapolated to juvenile stages and should be further investigated across different growth stages in future studies.

Taken together, the present findings emphasize the consistent response of AP7.5, maintaining productive and economic performance comparable to the Control across successive production stages and experimental scales, while commercial-size shrimp fed this formulation also showed a comparatively stable sensory profile and lower melanosis after seven days of refrigerated storage. Although the mechanisms underlying this post-harvest response remain unresolved, these findings indicate that dietary ingredient selection may influence both productive performance and subsequent product stability. By integrating biological performance, economic feasibility, post-harvest quality, and pilot-scale validation, the present study contributes to narrowing the gap between experimental low FM feed formulation and its practical application under production-relevant BFT conditions.

## 5. Conclusions

The present study demonstrates that AP7.5, formulated by reducing fishmeal from 15% to 7.5% through a complementary combination of animal- and plant-derived protein sources, represents a scalable low FM strategy for *Penaeus vannamei* cultured under BFT conditions. The formulation maintained growth and feed utilization comparable to the Control during grow-out and showed a consistent response across juvenile and grow-out phases when transferred to pilot large-scale conditions, supporting its nutritional robustness across production stages and scales. This productive consistency was accompanied by economic performance comparable to the Control, indicating that FM reduction could be achieved without compromising economic viability. Post-harvest quality was also maintained, with AP7.5-fed shrimp showing a comparatively stable sensory profile and lower panel-assessed melanosis after seven days of refrigerated storage without antimelanosis treatment. Although the mechanisms underlying this response remain to be elucidated, the results suggest that dietary ingredient selection may influence subsequent product stability. Overall, AP7.5 provides a practical and production-relevant strategy for reducing FM dependence while maintaining biological performance, economic feasibility, and post-harvest quality in BFT shrimp aquaculture.

## Figures and Tables

**Table 1:** Formulation and proximate composition of experimental diets (g/kg dry basis).

	Juvenile Diet (38% CP)			Grow-Out Diet (34% CP)	
	AP7.5	Control	A7.5	AP7.5	Control
**Feed ingredients (g/kg dry basis)**					
Fishmeal	75	150	75	75	150
Wheat	358	334	466	422	430
Soy	163	210		148	166
Porcine hemoglobin	20		67	15	
Meat meal	80		245	68	
Wheat hydrolyzed meal	55	50		40	35
Soy hydrolyzed protein	55	50		40	35
Potato protein	55	50		40	35
Lithonutri Seaweed	50	50	50	50	50
Fish oil	28	20	28	28	20
Soy oil	26	46	34	39	49
Pre-mixed vitamins	10	10	10	10	10
Calcium phosphate	15	15	15	15	15
Soy Lecithin	10	5	10	10	5
**Proximate composition (%)**					
Crude Protein (CP)	38.7	39.2	32.3	33.8	33.8
Crude Lipids (CL)	9.4	9.9	9.9	9.9	10.1
Carbohydrates *	42.1	40.2	42.4	47.3	46.5
Ash	9.8	10.7	15.4	9.0	9.6
**€/kg**	1.41	1.41	1.30	1.33	1.33

* Carbohydrates were calculated as: 100 − (CP + CL + Ash).

**Table 2:** Amino acid profile of the experimental diets, including essential and non-essential amino acid composition (g/100 g protein) in the different diets tested.

	Juvenile Diet (38% CP)		Grow-Out Diet (34% CP)		
	AP7.5	Control	A7.5	AP7.5	Control
**Essential AA (EAA)**					
Arginine (ARG)	5.90	5.80	5.79	5.66	5.72
Histidine (HIS)	2.39	2.28	3.14	2.40	2.20
Isoleucine (ILE)	3.94	4.73	3.03	4.26	4.61
Leucine (LEU)	8.33	8.33	8.76	8.33	8.08
Lysine (LYS)	5.22	5.50	4.85	5.32	5.68
Methionine (MET)	1.29	1.21	1.61	1.74	2.22
Phenylalanine (PHE)	5.12	5.26	4.77	5.18	5.10
Threonine (THR)	4.02	4.31	3.78	4.10	4.23
Valine (VAL)	5.38	5.27	5.65	5.14	5.08
**Non-essential AA (NEAA)**					
Alanine (ALA)	5.48	4.83	7.37	5.22	4.98
Aspartic acid (ASP)	9.54	9.78	8.68	9.24	9.70
Cysteine (CYS)	0.91	0.82	1.11	1.37	1.49
Glutamic acid (GLU)	20.30	21.54	16.10	20.59	21.00
Glycine (GLY)	6.65	5.02	10.08	5.89	4.87
Proline (PRO)	7.29	6.73	8.14	7.32	6.63
Serine (SER)	5.11	5.30	4.75	5.02	4.99
Tyrosine (TYR)	3.15	3.27	2.40	3.27	3.43
EAA	41.57	42.71	41.37	42.12	42.91
NEAA	58.43	57.29	58.63	57.88	57.09
EAA/NEAA	0.71	0.75	0.71	0.73	0.75
EAA/Total AA	0.42	0.43	0.41	0.42	0.43

**Table 3:** Growth performance of *P. vannamei* cultured using different feed diets (mean ± SE; *n* = 3). The values within the same column with different superscripts are significantly different at (*p* < 0.05).

**Medium Scale**								
**Treatment**	**Initial Weight (g)**	**Final Weight (g)**	**SGR (%/Day)**	**FI (g/100 g·day)**	**Apparent** **FCR**	**PIR**	**PER**	**Survival (%)**
A7.5	4.34 ± 1.23	14.98 ± 0.29	1.65 ± 0.17 **^b^**	3.18 ± 0.51	1.98 ± 0.10 **^a^**	0.95 ± 0.15	1.70 ± 0.09	79.00 ± 5.29
AP7.5	4.98 ± 1.13	16.92 ± 0.68	1.91 ± 0.19 **^a^**	2.41 ± 0.37	1.46 ± 0.10 **^b^**	0.74 ± 0.11	2.23 ± 0.17	79.33 ± 2.33
Control	4.47 ± 1.71	17.00 ± 0.60	2.06 ± 0.35 **^a^**	2.61 ± 0.37	1.48 ± 0.02 **^b^**	0.81 ± 0.12	2.17 ± 0.03	81.50 ± 5.50
**Final large-scale**								
**Treatment**	**Initial weight (g)**	**Final weight (g)**	**SGR (%/day)**	**FI (g/100 g·day)**	**FCR**	**PIR**	**PER**	**Survival (%)**
AP7.5	0.91 ± 0.03	9.76 ± 0.35	3.82 ± 0.06	2.83 ± 0.12	1.08 ± 0.05	1.00 ± 0.04	2.61 ± 0.15	84.67 ± 2.91
Control	0.91 ± 0.03	9.45 ± 0.07	3.78 ± 0.01	2.96 ± 0.01	1.13 ± 0.01	1.03 ± 0.01	2.49 ± 0.01	83.67 ± 2.03

**Table 4:** Economic conversion ratio (ECR) and Economic profit index (EPI) for the dietary treatments evaluated in the medium-scale grow-out and pilot large-scale validation trials (mean ± SE, *n* = 3). The values within the same column with different superscripts are significantly different at (*p* < 0.05).

Trial	Diet	ECR (€/kg Shrimp Biomass Produced)	EPI (€/kg Biomass)
**Medium-scale Grow-out**			
(34% CP) **	A7.5	2.58 ± 0.13 **^a^**	
	AP7.5	1.95 ± 0.14 **^b^**	
	Control	1.97 ± 0.03 **^b^**	
**Pilot large-scale validation**			
(38%CP) *	AP7.5	1.39 ± 0.06	
	Control	1.37 ± 0.02	
(34% CP) **	AP7.5	1.52 ± 0.20	
	Control	1.85 ± 0.05	
**Average large-scale** production ***	AP7.5	1.46 ± 0.13	3.75 ± 0.07
	Control	1.61 ± 0.01	3.65 ± 0.02

* 38% CP diet used during the juvenile diet of the pilot large-scale validation trial. ** 34% CP diet used during the grow-out phase. *** Average ECR calculated for the complete pilot large-scale validation trial.

**Table 5:** Protein and energy retention efficiency (%) of *P. vannamei* cultured using different tested feeds (mean ± SE, *n* = 3), (A7.5, AP7.5 and Control) during the medium-scale grow-out trial.

	Protein Retention (%)	Energy Retention (%)
A7.5	35.48 ± 3.08	24.54 ± 2.18
AP7.5	49.07 ± 5.37	31.04 ± 4.00
Control	47.93 ± 2.90	32.91 ± 4.32

**Table 6:** Proximate composition of shrimp and biofloc at the initial and final sampling points of the medium-scale grow-out trial under the different dietary treatments (A7.5, AP7.5 and Control) (mean ± S.E, *n* = 3). The (*) symbol refers to differences between initial and final sampling times (*p* < 0.05). DM (%): dry matter, Ashes (%), CP: crude protein, GE (MJ/kg): gross energy, and OM (%): Organic matter.

Sample		DM	Ashes	CP	GE
**Shrimp**	Initial	20.85 ± 0.28	15.17 ± 0.40	69.50 ± 0.39	18.28 ± 0.13
	A7.5	24.61 ± 0.97 *	13.18 ± 2.07	76.78 ± 1.06 *	21.33 ± 0.07 *
	AP7.5	25.80 ± 0.78 *	12.69 ± 1.61	75.21 ± 0.91 *	21.28 ± 0.16 *
	Control	25.93 ± 0.35 *	11.73 ± 2.18	76.69 ± 1.37 *	21.87 ± 0.32 *
		**OM**	**Ashes**	**CP**	**GE**
	Initial	44.19 ± 0.08	50.03 ± 0.04	23.27 ± 0.25	10.84 ± 0.10
**Biofloc**	A7.5	36.02 ± 0.97 *	57.02 ± 0.86 *	19.66 ± 0.80	9.59 ± 0.18
	AP7.5	39.50 ± 1.50 *	53.04 ± 0.96 *	20.75 ± 1.23	10.36 ± 0.36
	Control	41.40 ± 0.37	51.1 ± 2.16	22.09 ± 1.43	10.95 ± 0.02

**Table 7:** Integrated comparison of key performance indicators for AP7.5 relative to the Control diet across the medium-scale grow-out and pilot large-scale validation trials (mean ± SE, *n* = 3).

Variable	Medium-Scale Grow-Out Trial				Pilot Large-Scale Validation Trial				Cross-Scale Out-Come
	AP7.5	Control	AP7.5/Control (%) *	Δ (%) **	AP7.5	Control	AP7.5/Control (%) *	Δ (%) **	
SGR (%/Day)	1.91 ± 0.19	2.06 ± 0.35	92.7	−7.3%	3.82 ± 0.06	3.78 ± 0.01	101.1	+1.1%	Comparable growth response
Apparent FCR	1.46 ± 0.10	1.48 ± 0.02	98.6	−1.4%	1.08 ± 0.05	1.13 ± 0.01	95.6	−4.4%	Comparable feed efficiency
Survival (%)	79.33 ± 2.33	81.50 ± 5.50	97.3	−2.7%	84.67 ± 2.91	83.67 ± 2.03	101.2	+1.2%	Comparable survival
ECR (€/kg biomass gain)	1.95 ± 0.14	1.97 ± 0.03	99.0	−1.0%	1.46 ± 0.13	1.61 ± 0.01	90.7	−9.3%	Comparable/favorable economic response across scales
EPI (€/kg biomass)	-	-	-		3.75 ± 0.07	3.65 ± 0.02	102.7		Only evaluated in the pilot large-scale trial
Melanosis at day 7 (%)	25	63	39.7		-	-	-		Only evaluated in the medium-scale trial

* Relative to Control (%) was calculated as (AP7.5/Control) × 100 within each trial. ** Difference from Control was calculated as [(AP7.5 − Control)/Control] × 100 within each trial. Positive values indicate higher values for AP7.5, whereas negative values indicate lower values. For apparent FCR and ECR, negative values represent a more favorable response.

## Data Availability

The original data presented in the study are openly available in institutional repository RiuNet (Universitat Politècnica de València) at https://riunet.upv.es/handle/10251/239034 (accessed on 6 September 2026).

## Supplementary Materials

The following supporting information can be downloaded at https://www.mdpi.com/article/10.3390/ani16182852/s1. Annex SI: Sensory quality evaluation of farmed shrimp. Annex SII: Figure S1: Visual scale for assessing the presence of melanosis in raw and steamed *P. vannamei* from 0 to 100% corresponding to (A) to (F) respectively. Annex SIII: Table S1. Summary of repeated measures ANOVA for body weight evolution and phase specific growth rate (SGR during the pilot large scale validation trial. Table S2. ECR (€/kg biomass gain) sensitivity test to FM price during medium scale grow-out trial (Mean ± SE, *n* = 3). Table S3. ECR (€/kg biomass gain) sensitivity test to FM price during pilot large scale validation trial (Mean ± SE, *n* = 3). Table S4. EPI (€/kg biomass) sensitivity test to FM and shrimp price during pilot large scale validation trial (Mean ± SE, *n* = 3). Table S5. Water quality parameters in both experimental phases for *P. vannamei* cultivation with different diets during the grow-out stage and scale-up (mean ± S.E; *n* = 3). The values within the same column with different superscripts are significantly different at (*p* < 0.05). *Kruskal-wallis* test and *Bonferroni* post hoc test.

## Abbreviations

The following abbreviations are used in this manuscript:

AA	Amino acids
ALA	Alanine
ARG	Arginine
ASP	Aspartic acid
BFT	Biofloc technology
C	Carbon
CL	Crude lipid
CP	Crude protein
CYS	Cysteine
DM	Dry matter
EAA	Essential AA
ECR	Economic conversion ratio
EPI	Economic profit index
FCR	Feed conversion ratio
FI	Feed intake
FM	Fishmeal
GE	Gross energy
GLU	Glutamic acid
GLY	Glycine
HIS	Histidine
ILE	Isoleucine
LEU	Leucine
LYS	Lysine
MET	Methionine
N	Nitrogen
NEAA	Non-essential AA
OM	Organic matter
PCA	Principal Component Analysis
PER	Protein efficiency ratio
PHE	Phenylalanine
PIR	Protein intake ratio
PL	Post-larvae
PPO	Polyphenol oxidase
PRO	Proline
QDA	Quantitative descriptive analysis
SBM	Soybean meal
SE	Standard error
SER	Serine
SGR	Specific growth rate
THR	Threonine
TSS	Total suspended solids
TYR	Tyrosine
UNIZAR	Universidad de Zaragoza
UPV	Universitat politècnica de València
VAL	Valine
