# Supplementary material for: Growth Performance, Product Quality and Cost-Effectiveness of Alternative Protein Sources Replacing Fishmeal in Penaeus vannamei Reared in Biofloc Systems: Medium- and Large-Scale Studies
Source: Animals (Basel). 2026 Sep 10;16(18):2852. doi: 10.3390/ani16182852 (PMC13603790; doi:10.3390/ani16182852)
Supplement: Supplementary file 1 [file animals-16-02852-s001.zip › animals-4465818-supplementary.pdf]

SUPPLEMENTARY MATERIAL

ANNEX SI

GAB Grupo de Acuicultura y Biodiversidad

Instituto Universitario de Investigación Mixto Agroalimentario de Aragón Universidad Zaragoza

SENSORY QUALITY EVALUATION OF FARMED SHRIMP

With the shrimp laying down on a plate, analyze the following aspects:

Blackspot defect (melanosis progression)

0 2 4 6 8 10

Red-orange color intensity (aspect)

Faint Intense

Brightness intensity in the shell

Faint Intense

Shrimp evaluation (AROMA)

Characteristic steamed shrimp aroma

Absent Intense

Marine aroma

Absent Intense

Freshly cut grass aroma

Absent Intense

Cucumber aroma

Absent Intense

Iodine odor

Absent Intense

Acetic odor

Absent Intense

Rancid odor

Absent Intense

Spoilage odor

Absent Intense

Once the whole cooked shrimp has been evaluated, the shell is removed for meat evaluation.

Shrimp evaluation at the mouth (TEXTURE)

Firmness (first bite with incisors)

Soft Firm

Fibrosity (in mouth, during mastication)

Nothing Much

Shrimp evaluation at the mouth (FLAVOR)

Remember to take deep breaths prior to each evaluation.

Characteristic flavor to shrimp (tasting and breathing with the sample)

Absent Intense

Wild flavor (salty-iodized)

Absent Intense

Metallic flavor

Absent Intense

Sweet flavor

Absent Intense

Astringent flavor

Absent Intense

Sour flavor

Absent Intense

Residual flavor (Immediately after swallowing the sample)

Absent Intense

ANNEX SII

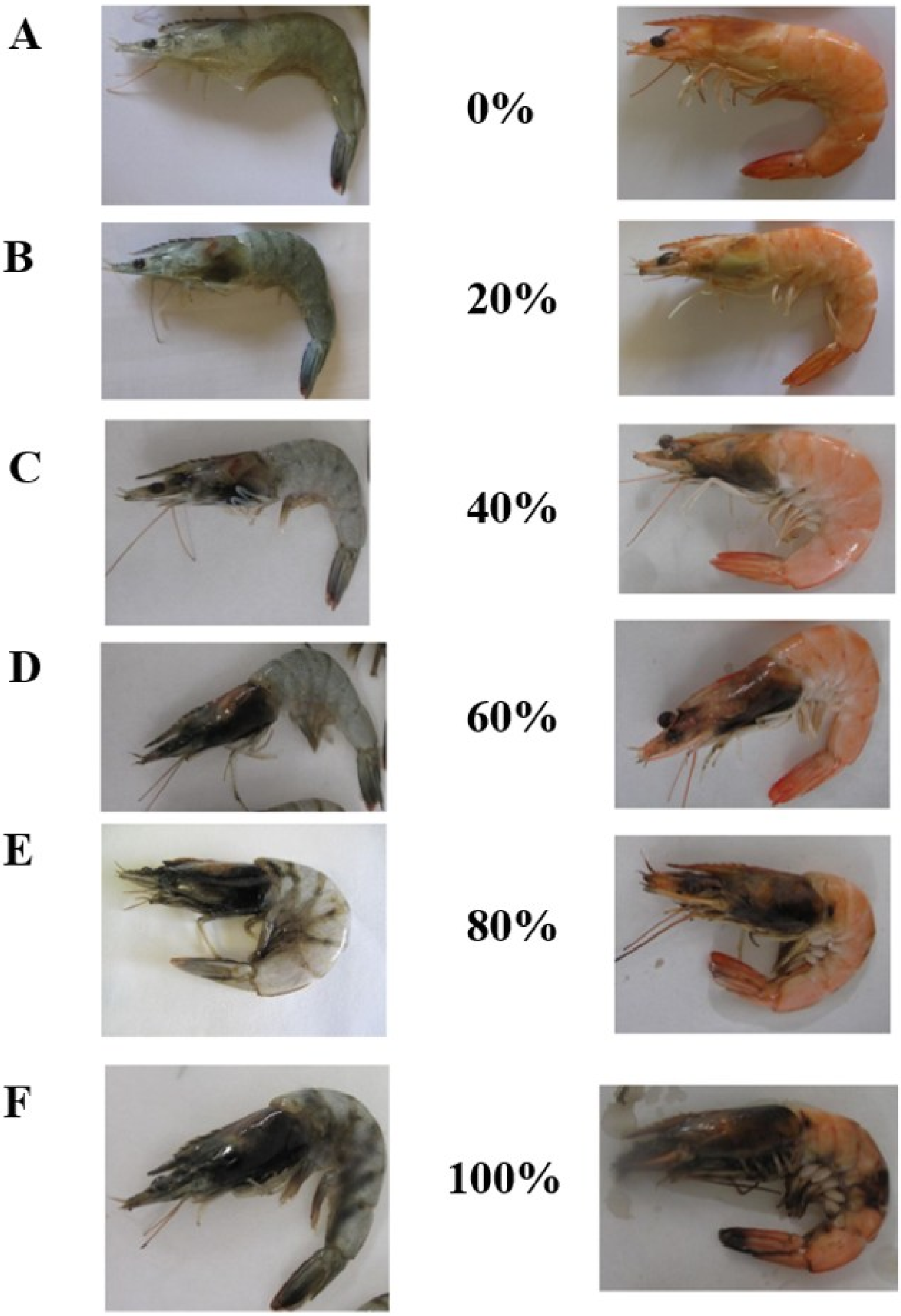

Figure S1: Visual scale for assessing the presence of melanosis in raw and steamed *P. vannamei* from 0 to 100%, corresponding to (A) to (F) respectively.

ANNEX SIII

Table S1. Summary of repeated measures ANOVA for body weight evolution and phase specific growth rate (SGR during the pilot large scale validation trial.

Response variable	Effect	df	F	p-value
Mean body weight	Diet	1, 4	0.02	0.89
	Time	1.17, 4.69 [1]	380.80	<0.001
	Diet x Time	1.17, 4.69 [1]	0.26	0.67
SGR	Diet	1, 4	1.20	0.334
	Phase	1, 4	515.68	<0.001
	Diet x Phase	1, 4	3.24	0.15

Sensitivity scenarios were generated by varying the baseline fishmeal price (1.39 €/kg) and shrimp sale price (5.22 €/kg) by ±20%. Prices of all other dietary ingredients and experimentally observed biological performance were kept constant

Table S2. ECR (€/kg biomass gain) sensitivity test to FM price during medium scale grow-out trial (Mean±SE, *n*=3)

FM price scenario	A7.5	AP7.5	Control
-20%	2.53±0.13	1.92±0.14	1.91±0.03
Base	2.57±0.13	1.95±0.14	1.97±0.03
+20%	2.62±0.13	1.98±0.14	2.03±0.03

Table S3. ECR (€/kg biomass gain) sensitivity test to FM price during pilot large scale validation trial (Mean±SE, *n*=3)

	Juvenile diet (38% CP)		Grow-out diet (34% CP)	
FM price scenario	AP7.5	Control	AP7.5	Control
-20%	1. 37±0.06	1.32±0.02	1.50±0.20	1.79±0.05
Base	1.39±0.06	1.37±0.02	1.52±0.20	1.85±0.05
+20%	1.41±0.06	1.41±0.02	1.54±0.20	1.91±0.05

TableS4. EPI (€/kg biomass) sensitivity test to FM and shrimp price during pilot large scale validation trial (Mean±SE, *n*=3)

FM price scenario	Shrimp sale scenario	AP7.5	Control
-20%	-20%	2.73±0.07	2.66±0.02
-20%	Base	3.77±0.07	3.70±0.02
-20%	+20%	4.82±0.07	4.75±0.02
Base	-20%	2.71±0.07	2.61±0.03
Base	Base	3.75±0.07	3.65±0.03
Base	+20%	4.79±0.07	4.70±0.03
+20%	-20%	2.68±0.07	2.56±0.03
+20%	Base	3.73±0.07	3.61±0.03
+20%	+20%	4.77±0.07	4.65±0.03

Table S5. Water quality parameters in both experimental phases for *P.vannamei* cultivation with different diets during the grow-out stage and scale-up (mean ± S.E; *n*=3). The values within the same column with different superscripts are significantly different at (p < 0.05). *Kruskal-wallis* test and *Bonferroni* post hoc test.

Grow-out Stage	A7.5	AP7.5	Control
pH	7.95±0.02	8.00±0.01	8.00±0.01
Oxygen (mg/L)	5.92±0.03 ^{b}	6.03±0.04 ^{a}	5.98±0.04 ^{ab}
Salinity (g/L)	20.06±0.02 ^{b}	20.22±0.06 ^{ab}	20.17±0.08 ^{ab}
Temperature (ºC)	28.00±0.05	29.31±1.38	27.90±0.07
Ammonia (mg/L)	0.05±0.01	0.06±0.01	0.07±0.02
Nitrite (mg/L)	0.38±0.10	0.84±0.22	0.63±0.19
Nitrate (mg/L)	74.40±30.00	63.01±25.10	60.04±30.38
Phosphorus (mg/L)	4.34±0.64	5.41±1.09	4.16±0.49
SSTs (mg/L)	448.74±38.76	369.14±32.53	397.98±37.40
Alkalinity (mg/L)	213.87±4.98 ^{a}	185.98±2.89 ^{b}	187.53±3.39 ^{b}

**Final<br>Scale-up**	**AP7.5**	**Control**
pH	7.77±0.02	7.73±0.02
Oxygen (mg/L)	6.12±0.02	6.14±0.02
Salinity (g/L)	20.34±0.03	20.29±0.04
Temperature (ºC)	28.31±0.08	28.38±0.09
Ammonia (mg/L)	0.08±0.01	0.09±0.02
Nitrite (mg/L)	0.06±0.01	0.07±0.02
Nitrate (mg/L)	49.29±12.52	46.42±11.45
Phosphorus (mg/L)	4.77±0.62	4.69±0.40
SSTs (mg/L)	306.90±17.66	301.45±17.28
Alkalinity (mg/L)	212.33±8.84	200.87±8.04
